# From vivarium to NAM-centred laboratory: A practical framework for managing infrastructure, expertise and organisational transition

**DOI:** 10.1016/j.namjnl.2026.100129

**Published:** 2026-09-07

**Authors:** Clive Roper, Michael D. Coleman

**Affiliations:** a Roper Toxicology Consulting Limited, 6 St Colme Street, Edinburgh, EH3 6AD, UK; b School of Medicine, Pharmacy and Biosciences, Aston University, Aston Triangle, Birmingham, B4 7ET, UK

**Keywords:** Knowledge retention, Workforce development, Change management, Infrastructure transition, Business sustainability, New Approach Methodologies, Vivarium

## Abstract

Political, Regulatory and Scientific developments are propelling the use of New Approach Methodologies (NAMs) across regulatory toxicology and drug development, creating challenges for organisations operating animal-testing facilities. High clinical attrition rates driven by lack of efficacy and toxicity continue to stimulate interest in more human-predictive approaches. The FDA Modernization Acts and UK and EU initiatives to phase out animal testing are also influencing future testing strategies. Universities, pharmaceutical companies and contract research organisations (CROs) have made substantial investments in facilities, equipment, support services and highly skilled personnel. Increasing adoption of NAMs forces existing infrastructure redevelopment, whilst preserving capital investment and retaining/repurposing scientific expertise. A practical framework is presented for organisations transitioning from animal-based testing towards NAM-centred operations, addressing infrastructure redevelopment, workforce transition, expertise retention and capital investment preservation. Infrastructure requirements, support services, workforce development, business sustainability and role-specific staff retraining are examined. Retaining institutional knowledge and recognising the continuing value of technical, scientific and regulatory expertise developed through animal-testing programmes as organisations transition towards NAMs are prioritised. Contexts of Use (CoUs) and Adverse Outcome Pathways (AOPs) help connect historical animal-study knowledge with future NAM applications. Successful transition is presented as a managed organisational process involving people, infrastructure, knowledge and investment rather than simply the replacement of one experimental model with another.

## Highlights

• Practical framework for transition to NAM-centred laboratories.
• Existing infrastructure can support redevelopment for NAM adoption.
• Workforce retraining helps retain expertise and institutional knowledge.
• CoUs and AOPs connect historical animal data with future NAM use.
• Successful transition requires organisational, not just scientific, change.

## Introduction

The transition from animal-based laboratories to New Approach Methodologies (NAM)-centred laboratories is not a simple substitution of one husbandry system for another. However, many existing competencies within animal research organisations remain highly relevant, provided the transition is managed through role-specific training, realistic infrastructure planning and integration of regulatory, analytical and biological expertise.

Over the past two decades, scientific policy has been moving inexorably towards the adoption of NAMs with the publications of the FDA Modernization Act (H.R.2565, 2022; Stewart et al., 2023), FDA Modernization Act 3.0 (H.R.2821, 2022), the UK Government strategy for replacing animals in science (UK Government, 2025), and the EU roadmap towards phasing out animal testing for chemical safety assessments (European Commission, 2026). Regulatory agencies have also highlighted both the opportunities and challenges associated with incorporating NAMs into nonclinical safety assessment and drug development programmes (Avila et al., 2020; Avila et al., 2023). Stakeholders have also identified workforce development, training, organisational change and infrastructure requirements as important factors influencing successful adoption of NAMs in practice (Sewell et al., 2024; Ouedraogo et al., 2025).

Consequently, universities, pharmaceutical companies and CROs operating animal facilities face strategic decisions regarding future infrastructure, workforce planning, capital investment and long-term business sustainability. The debate often focuses on scientific replacement and regulatory acceptance, but less attention is paid to how organisations can practically manage the transition of facilities, support services, institutional knowledge and the highly skilled staff responsible for generating, interpreting and reporting animal study data.

Detailed discussions of NAMs, Adverse Outcome Pathways (AOPs) and Contexts of Use (CoU) are beyond the scope of this article and have been reviewed elsewhere (Ankley et al., 2010; OECD, 2017; Sewell et al., 2024; LaFollette et al., 2025; LaFollette et al., 2026; Macmillan et al., 2026; Filipova et al., 2026). However, an understanding of these concepts remains important because the intended application of a NAM ultimately determines the facilities, expertise, support services and organisational capabilities required to support its implementation.

## Adoption of NAMs - A situation in Flux

Of course, in order for the gradual transition from a primarily animal-centric, towards a more human platform-sourced regulatory position to progress, it must be aligned with the type of regulatory questions NAMs are expected to answer. One of the most powerful drivers of NAM adoption, is essentially self-fulfilling, as the use of NAMs in drug discovery has revealed a raft of novel therapeutic approaches ranging from monoclonals to oligonucleotides and protein degradation systems that are exclusively human in terms of their targets (Wu et al., 2026), so reinforcing the value of the non-animal experimental platforms where they were discovered. In addition, both efficacy and toxicity data from animal platforms will have little or no relevance to patients with these agents. In other areas, another scientific driver behind the growing interest in NAMs is the continuing challenge of translating findings from existing animal-based preclinical models into successful human outcomes. Historically, analyses of chronically high drug development attrition demonstrate that the principal causes of failure are lack of clinical efficacy (40-50%), unmanageable toxicity (*ca* 30%), poor drug-like properties (10-15%), and commercial or strategic considerations (*ca* 10%) (Sun et al., 2022; Filipova et al., 2026). Considering clinical efficacy, the most extreme situation is probably the inability of the pharmaceutical industry to develop successful anti-Alzheimer’s drugs (>99% clinical failure). This can be laid squarely at the door of the inability of existing animal models to even tangentially model the human course of the condition (Veening-Griffioen et al., 2019). Even in other therapeutic areas where animal platforms have been reported to be more successful, in terms of efficacy determination, such as anticancer drug development (Guo et al., 2024), human toxicity predictiveness in the same area yielded by animal models was poor (Atkins et al., 2020). This situation has become blurred somewhat with the growing adoption of non-mammalian animal models in efficacy and toxicity studies, such as *Caenorhabditis elegans* (roundworms) and *Danio r*e*rio* (zebrafish; Hunter, 2023). However, candidate drug failure caused by toxicity is also recognised to be due to animal models’ inability to reflect human genetic variation (Atkins et al., 2020). Indeed, it can be argued that the huge investment in animal support technology and expertise over the past few decades has delivered sub-optimal returns, in the sense of providing the preclinical basis for the marketing of therapeutically effective, safe and commercially successful medicines.

It is anticipated that current high candidate drug attrition rates are very likely to fall as more faithful human-sourced platforms are gradually adopted, based around advanced systems such as iPSCs, organoid and organ-on-a-chip models (Wu et al., 2026). These models and myriad other NAMs have the potential to dramatically reconfigure and facilitate successful pharmaceutical drug development over the next decade (Wu et al., 2026).

## Actively facilitating the adoption of NAMS

Whilst NAM usage has gathered momentum in scientific, regulatory and societal terms, many practical questions remain over the mechanics of this process, not least the large pool of existing animal-test expertise in commercial and academic environments. How this expertise can be reconfigured is an important consideration during the gradual transition to NAM-based testing because personnel involved in animal studies understand the strengths and weaknesses of the *in vivo* models on which current regulatory decisions are based. We postulate that this knowledge will be essential for the effective development, application and interpretation of NAMs. This is because animal tests generate multiple outputs, such as clinical signs, biomarkers, organ weight changes and pathology findings. Those outputs are used in regulatory decision-making. Understanding how those outputs are generated, interpreted and used within regulatory submissions is, therefore, likely to be an important consideration when planning transition from animal-based testing to NAM-centred operations. The implementation, interpretation and development of NAMs will be expedited considerably if organisations understand what endpoints animal studies were historically being applied towards and how those data contributed to regulatory decision-making. CoU and AOP concepts help convert those historical animal outputs into mechanistic and regulatory questions that can be addressed by NAMs. In this respect, CoUs and AOPs act as translational tools between traditional animal studies and future NAM-based approaches.

## Learning from existing animal studies

Effective transition from animal-based testing to NAM-centred operations requires an understanding of the information generated by existing regulatory studies, the scientific questions they were designed to answer, and how those data have historically been used in regulatory decision-making. Animal inhalation studies, for example, integrate diverse disciplines including exposure science, pathology, immunology, bioanalysis and analytical chemistry. Understanding these endpoints and their relevance to human health is essential when designing replacement strategies and when planning how existing expertise, facilities and support services can contribute to future NAM-centred testing. A practical example is the OECD case study on chlorothalonil inhalation toxicity (Roper et al., 2022), which used a combination of *in chemico, in vitro* and *in silico* approaches in place of a traditional animal study. A commercially available human airway model (MucilAir™) was combined with exposure assessment and computational modelling to support human risk assessment, resulting in a waiver of a 90-day rat inhalation study by the US EPA. Successful NAM implementation depends not only on new technologies, but also on expertise gained from animal studies. Knowledge of toxicology, pathology, exposure assessment, bioanalysis and regulatory requirements remains essential for identifying appropriate endpoints, interpreting results and establishing the CoU for new methods. This expertise represents a valuable organisational asset that can be retained and repurposed during transition to NAM-centred testing, rather than being viewed solely as expertise associated with historical animal-testing programmes. The challenge for organisations is, therefore, not simply adoption of new technologies, but ensuring that existing scientific, technical and regulatory knowledge is effectively integrated into future testing strategies.

## Infrastructure, resources and workforce transition

The transition from animal-based testing to NAMs is often presented primarily as a scientific challenge. However, it is equally an organisational, infrastructure and workforce challenge. If animal testing decreases over time, organisations must decide whether existing facilities and infrastructure become stranded assets and how existing scientific expertise, operational capability and institutional knowledge can be preserved and adapted to support future testing strategies. These decisions require practical consideration of facilities, investment, support services, workforce development and business sustainability. Barriers to NAM implementation extend beyond scientific validation and include technical, economic, organisational, educational and cultural considerations (Sewell et al., 2024; Čavoški et al., 2024).

## Infrastructure and Facilities

Although there are important differences between animal-based and cell-based laboratories, many elements of existing infrastructure may continue to provide value within future NAM-centred operations. A vivarium is already designed to operate under tightly controlled environmental conditions, including temperature, humidity and monitoring systems. These features are also required for many cell culture and microphysiological system laboratories. Consequently, existing facilities may provide a strong starting point for redevelopment and integration into future NAM-centred operations, rather than requiring complete replacement (Leung et al., 2022; Mathisen et al., 2024).

Conversion of a vivarium into an *in vitro* testing facility should not be viewed as a simple "plug-and-play" exercise. Whilst existing environmental controls and building infrastructure may provide a strong starting point, substantial refurbishment and additional investment are typically required, including HEPA-filtered air handling systems, sterile laminar-flow workstations, cell culture incubators, gas supply systems for carbon dioxide and associated monitoring equipment. Advanced microphysiological systems and organ-on-chip technologies require standardised and highly controlled operating environments to ensure reliable performance and regulatory confidence (Leung et al., 2022; Ramadan and Zourob, 2020; Piergiovanni et al., 2021). Cell culture facilities also require rigorous contamination-control procedures, with incubators representing a common source of microbial contamination if not properly maintained. Extensive cleaning, refurbishment and validation would, therefore, be necessary before existing animal facilities could support routine cell culture operations.

The financial investment associated with this transition can be substantial. Advanced cell and tissue culture platforms, organoids, organ-on-chip systems and associated equipment are expensive. This does not include the costs associated with test system licensing arrangements and royalty payments, staff training, proficiency testing and ongoing operational support. Aside from this, a substantial proportion of the environmental control, building infrastructure and support services already present within animal testing facilities may still be applicable. This provides an opportunity to preserve previous capital investment whilst reducing the scale of redevelopment required for transition to NAM-centred testing (Leung et al., 2022; Farhang Doost and Srivastava, 2024).

## Support services

One frequently overlooked aspect of the transition is that many support functions remain essential irrespective of whether data originate from animals or from human-based *in vitro* models. Analytical chemistry, bioanalysis, pathology, immunology, quality assurance, data management and reporting all continue to be required (Turner et al., 2023). In many organisations the analytical laboratories generating the data may require only modest adaptation to support new sample types (LaFollette et al., 2025). For example, analytical methods developed to measure biomarkers, drug concentrations or biochemical changes in animal studies can often be adapted to support equivalent measurements in cell culture systems, organoids or microphysiological systems. One early illustration of such an adaptation was the successful application of the mainstays of animal brain slice neurophysiological analysis, patch clamping and fluorescent calcium imaging, to *in vitro* human neuronal/astrocytic cell cultures grown on glass slides (Hill et al., 2012).

While some technical modifications may be necessary, the fundamental scientific disciplines and infrastructure remain highly relevant. As a result, much of the analytical capability developed to support animal testing can continue to provide value within a NAM-centred organisation, creating opportunities to retain expertise, preserve existing capability and reduce the extent of organisational change required during transition (LaFollette et al., 2025).

## Artificial intelligence and data integration

Artificial Intelligence (AI) is likely to become an important component of future NAM-centred organisations. Advanced NAM platforms, including organoids, microphysiological systems, high-content imaging technologies and multi-omics approaches, can generate large and complex datasets that may be difficult to interpret using conventional analytical approaches alone. AI and machine-learning methods may assist by integrating multiple data streams, identifying mechanistic patterns, improving prediction of toxicological outcomes and facilitating interpretation of large-scale datasets derived from diverse experimental platforms. These methods have been particularly effective in areas such as human transcriptomics, facilitating the establishment of new biomarkers in disease, as well as tracking the impact of disease on gene expression (Cheng et al., 2024) and responses to novel therapeutics (Wu et al., 2026).

Beyond data analysis, AI may also support study design and implementation. By learning from historical datasets and previous experimental outcomes, AI-based tools could help identify potential limitations of NAM platforms, suggest complementary approaches and support selection of methods that are most appropriate for a specific CoU. Quality frameworks, such as the NC3Rs DRIVER Recommendations (NC3Rs, 2026), may also be incorporated into study design, study plan development and relevant standard operating procedures (SOPs), helping ensure that studies generate data suitable for transparent reporting and interpretation. This may improve resource allocation, reduce unnecessary experimentation and strengthen the overall evidential value of integrated testing strategies. However, these potential benefits must be balanced against recognised challenges. AI-derived outputs are dependent upon the quality, representativeness and transparency of the underlying data and algorithms. Bias, incomplete datasets, lack of explainability and over-reliance on automated outputs could undermine scientific confidence and regulatory acceptance if not appropriately managed. Consequently, AI should be viewed as a tool that complements, rather than replaces, scientific judgement, toxicological expertise and established validation principles. Therefore, organisations planning transition towards NAM-centred operations should consider development of data-science and AI literacy alongside investment in biological, engineering and regulatory expertise.

## Staff and expertise

The greatest asset within many animal testing organisations is not the facility itself, but the expertise, experience and institutional knowledge of the people working within it. Staff involved in animal studies possess experience in toxicology, study conduct, specimen collection, pathology, exposure assessment, data interpretation and regulatory requirements. This knowledge has considerable value when designing, validating and interpreting NAMs. The successful implementation of microphysiological systems and other advanced NAMs requires collaboration between technology developers, end users and regulators, highlighting the continuing importance of experienced scientific personnel (LaFollette et al., 2025).

Workforce transition should not be portrayed as a simple substitution of animal husbandry with cell culture or other NAM-based technologies. Different staff groups will require different levels of retraining. Workforce development and education have been identified as important factors influencing successful adoption of NAMs (Sewell et al., 2024; Čavoški et al., 2024).

Animal care technicians, for example, already possess highly transferable skills including attention to detail, adherence to procedures, environmental monitoring, record keeping and routine biological husbandry. With appropriate training in aseptic technique and contamination control, many could contribute to routine cell culture maintenance, laboratory operations and sample management. These skills may allow organisations to retain experienced personnel whilst reducing recruitment and training requirements during transition.

Similarly, technical staff experienced in animal sampling, sample preparation and storage may transition to operation of *in vitro* assays and analytical platforms. Routine analytical kits and biomarker measurements are often highly standardised and can be incorporated into new workflows through targeted practical training. This may enable organisations to redeploy experienced personnel into new operational roles whilst retaining practical laboratory capability and established quality standards.

Scientists and study directors bring a different but equally important contribution. Their understanding of toxicological endpoints, study design and regulatory expectations can help ensure that NAMs are developed to answer relevant regulatory questions rather than simply generating mechanistic data. This expertise may also help organisations avoid unnecessary redevelopment of testing strategies by retaining knowledge of why historical studies were performed, which endpoints informed regulatory decisions and where NAMs can provide equivalent or complementary information. As organisations increasingly adopt NAMs, successful transition is likely to depend on effective integration of expertise from both *in vivo* and *in vitro* disciplines rather than replacement of one knowledge base with another. Stakeholder engagement and clear understanding of CoU have been identified as key factors supporting regulatory acceptance of advanced NAM-based approaches. These considerations may also help organisations prioritise investments, training and implementation strategies during transition to NAM-centred operations (LaFollette et al., 2025).

Table 1 summarises the principal staff groups, their transferable expertise, potential NAM-transition pathways and key risks associated with poorly managed organisational transition.

**Table 1:** Transferable expertise, transition pathways and risks associated with transition to NAM-centred operations.

Staff Group	Existing Expertise and Value	NAM-Transition Pathway	Potential Risk if Transition is Poorly Managed
Animal care technicians	Husbandry discipline, observation, routine care, SOP adherence	Aseptic handling, routine culture maintenance, sample logistics, environmental monitoring	Overstating transition ease
Technical staff	Dosing, sampling, equipment use, and procedural consistency	Assay execution, sampling from *in vitro* systems, kit-based endpoints, contamination checks	Under-training or role ambiguity
Analytical chemistry/ bioanalysis staff	Method development, sample analysis, data quality	Continued support for *in vitro* exposure, dose confirmation, biomarker assays	Assuming no workflow changes
Pathology/ immunology staff	Endpoint interpretation, tissue/ cell response knowledge	Translation of histological and immunological endpoints into NAM endpoints, biomarkers and mechanistic readouts	Losing interpretive expertise
Study directors/ scientists	Study design, regulatory relevance, decision-making	NAM study design, CoU framing, AOP-based endpoint selection	Leaving NAMs disconnected from regulatory need
QA/ reporting/ archive staff	GLP-style documentation and traceability	Data integrity, validation documentation, audit trails for NAMs	Weak regulatory confidence

## A managed transition

The future adoption of NAMs should, therefore, not be viewed simply as the replacement of one set of experimental models with another. Rather, it represents a managed organisational transition involving redevelopment of existing infrastructure and facilities, investment in new technologies, retraining of staff, retention of valuable institutional knowledge, and preservation of existing scientific and operational capability. Organisations that successfully combine existing expertise with emerging technologies may be better positioned to adapt to future regulatory requirements and scientific advances. Organisations that plan proactively for infrastructure redevelopment, workforce transition and retention of institutional knowledge may be better positioned to adapt to future scientific, regulatory and commercial requirements. Similar conclusions have been reached by authors advocating system-level approaches to accelerate NAM adoption and overcome the scientific, organisational and cultural barriers associated with change (Sewell et al., 2024; Mathisen et al., 2024).

## Conclusions

The increasing adoption of NAMs is creating both scientific and organisational challenges for laboratories, universities, pharmaceutical companies and CROs, requiring strategic consideration of infrastructure, workforce development, investment and long-term organisational capability. Whilst regulatory and scientific developments are influencing future testing strategies, the practical implications for facilities, infrastructure, support services and staff have received comparatively less attention.

The transition from animal-based testing to NAM-centred operations can be viewed as an opportunity to reconfigure current organisational assets such as facilities, support services, quality systems, analytical capabilities and accumulated scientific, technical and regulatory expertise. Much of this infrastructure and expertise may remain valuable if appropriately redeveloped, retrained and integrated into future testing strategies.

Successful transition will depend upon understanding the purpose, regulatory context and historical contribution of existing animal studies, preserving institutional knowledge, and ensuring that expertise from both *in vivo* and *in vitro* disciplines is effectively combined. Decisions relating to infrastructure redevelopment, workforce planning, investment and implementation should, therefore, be considered together rather than as separate activities.

The framework presented here is intended to support practical planning for organisations considering transition towards NAM-centred testing. Organisations that retain and repurpose existing capabilities whilst investing in new technologies may be better positioned to adapt to future scientific, regulatory and commercial requirements. The framework presented here is intended to support those planning and implementation decisions, which it is anticipated, will ultimately yield better predictive preclinical data and lead to more effective and safer medicines in the future.

## Funding

This research did not receive any specific grant from funding agencies in the public, commercial, or not-for-profit sectors.

## CRediT authorship contribution statement

**Clive Roper:** Writing – review & editing, Writing – original draft, Investigation, Conceptualization, Methodology. **Michael D. Coleman:** Writing – review & editing, Conceptualization, Methodology.

## Declaration of competing interest

The authors declare that they have no known competing financial interests or personal relationships that could have appeared to influence the work reported in this paper.

## Data Availability

No data was used for the research described in the article.
